# Supplementary material for: Multi-Kingdom Gut Microbiome Interaction Characteristics Predict Immune Checkpoint Inhibitor Efficacy Across Pan-Cancer Cohorts
Source: Microorganisms. 2025 Nov 14;13(11):2595. doi: 10.3390/microorganisms13112595 (PMC12654497; doi:10.3390/microorganisms13112595)
Supplement: Supplementary file 1 [file microorganisms-13-02595-s001.zip › microorganisms-3700716-supplementary.pdf]

SUPPLEMENTARY INFORMATION CONTENTS

Supplementary Table

Supplementary Table S1. Comprehensive Demographic information of Cohorts

Supplementary Figures

Supplementary Fig. S1. Microbial diversity analysis from multi-kingdom microbiota between two subtypes

Supplementary Fig. S2. Clinicopathological characreristics of patients between two subtypes

Supplementary Fig. S3. Microbial diversity analysis across multi-kingdoms between responders and non-responders to ICI therapy

Supplementary Fig. S4. Distribution of SparCC correlation coefficients for different multi-kingdom combinations between two subtypes

Supplementary Fig. S5. Network with *Komagataella* between two subtypes

Supplementary Table S1. Comprehensive Demographic information of Cohorts

**Characteristic**	subtype C1	subtype C2
BMI	28.71 (± 5.50)<br>(n= 140)	29.30 (± 5.93)<br>(n= 241)
Antibiotics（No/Yes）	113/27	194/35
Sex（Female/Male）	45/95	59/182
Age（<65/>=65）	76/64	98/143

Perform an ANOVA on BMI, and perform Fisher's exact tests on Antibiotics, Sex, Age, and Therapy.

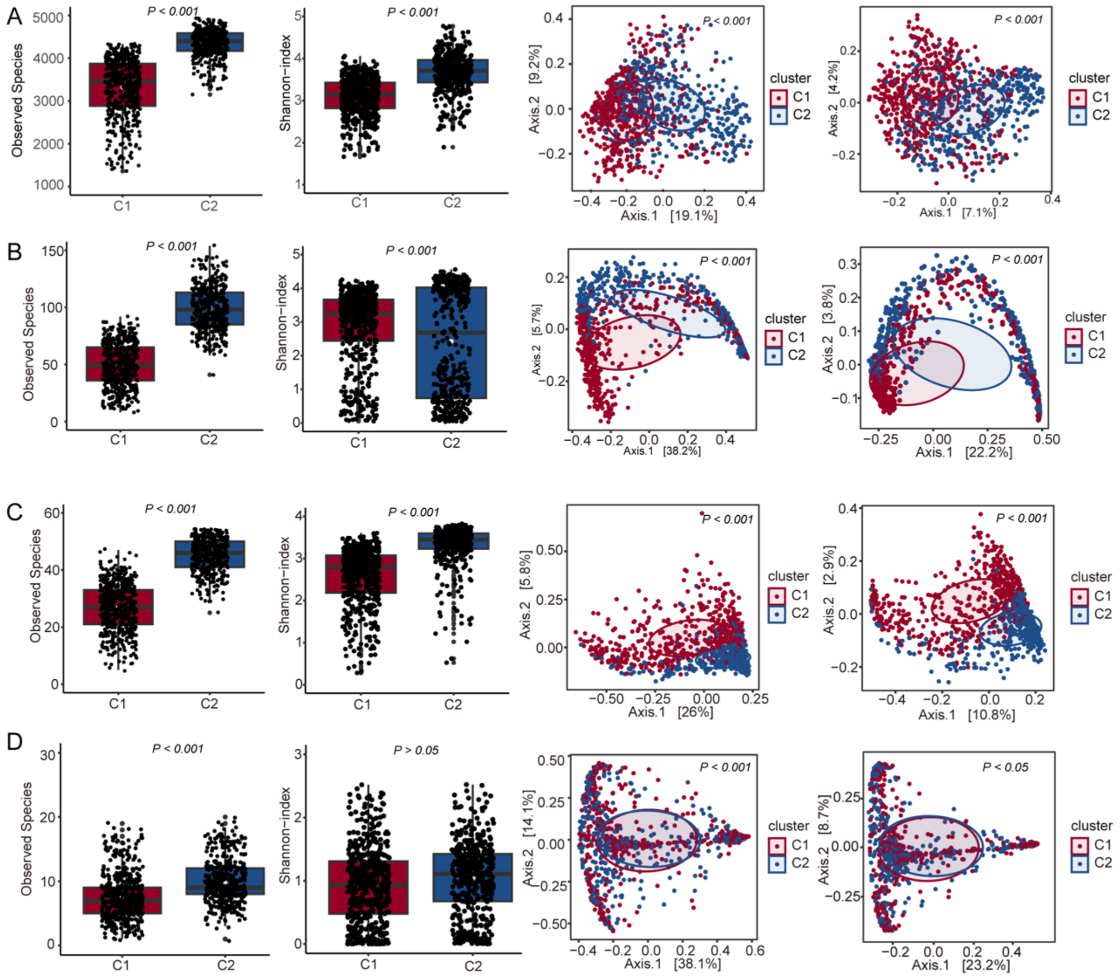

Supplementary Fig. S1. Microbial diversity analysis from multi-kingdom microbiota between two subtypes

Differences in alpha and beta diversity indices of bacteria (A), archaea (B), fungi (C), and viruses (D) among subtypes were analyzed. From left to right, the indices are alpha diversity (Observed Species, Shannon index) and beta diversity (PCoA based on Bray-Curtis and Jaccard). *P* value less than 0.001 or 0.05 were considered statistically significant.

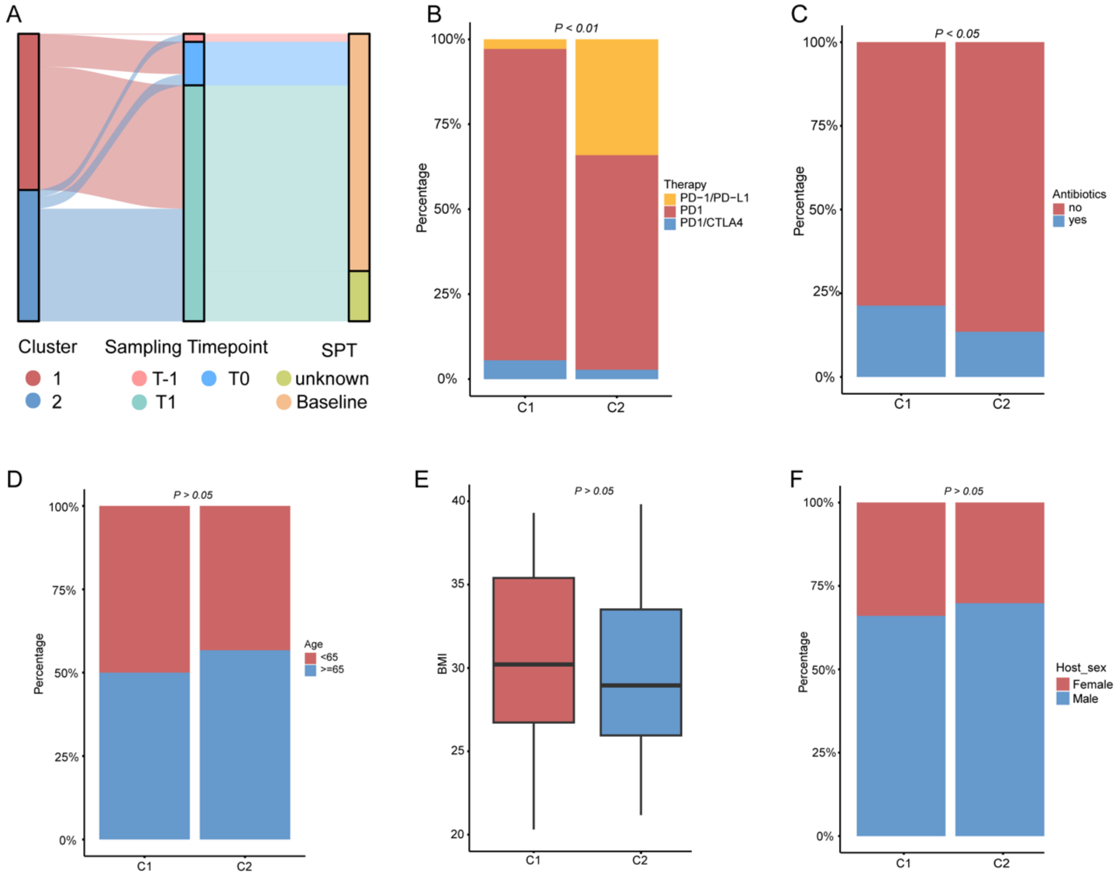

Supplementary Fig. S2. Clinicopathological characreristics of patients between two subtypes

(A) The strip plot displays the Sampling Timepoint and Sampling Processing Time (SPT) of samples within Clusters. (B-F) Differences in bacterial-fungal interactions, Therapy, Antibiotics, Age, BMI, and Host sex between subtypes C1 and C2 were analyzed. *P* value less than 0.01 or 0.05 was considered statistically significant.

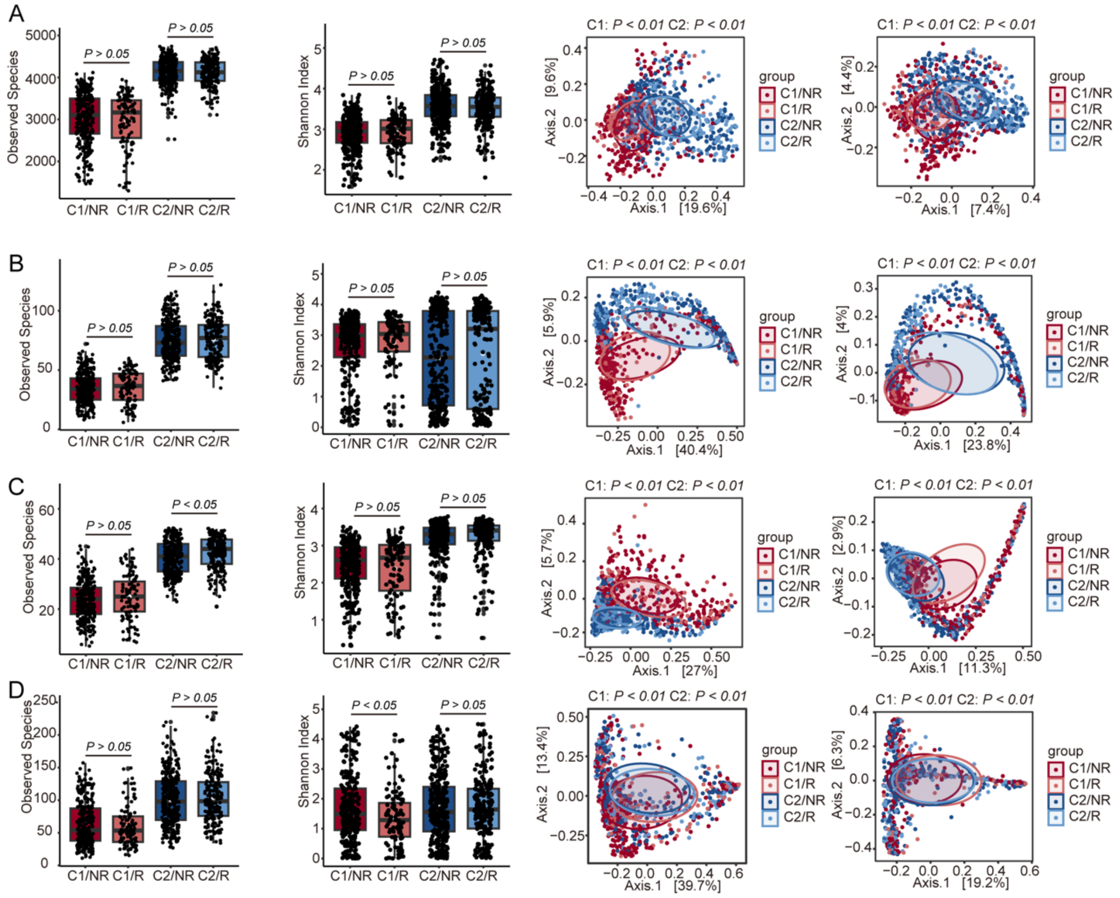

Supplementary Fig. S3. Microbial diversity analysis across multi-kingdoms between responders and non-responders to ICI therapy

Differences in alpha and beta diversity indices of bacteria (A), archaea (B), fu ngi (C), and viruses (D) between Responders and Non-Responders within subty pes. From left to right, the indices are alpha diversity (Observed Species, Shan non index) and beta diversity (PCoA based on Bray-Curtis and Jaccard). *P* val ue less than 0.01 or 0.05 were considered statistically significant.

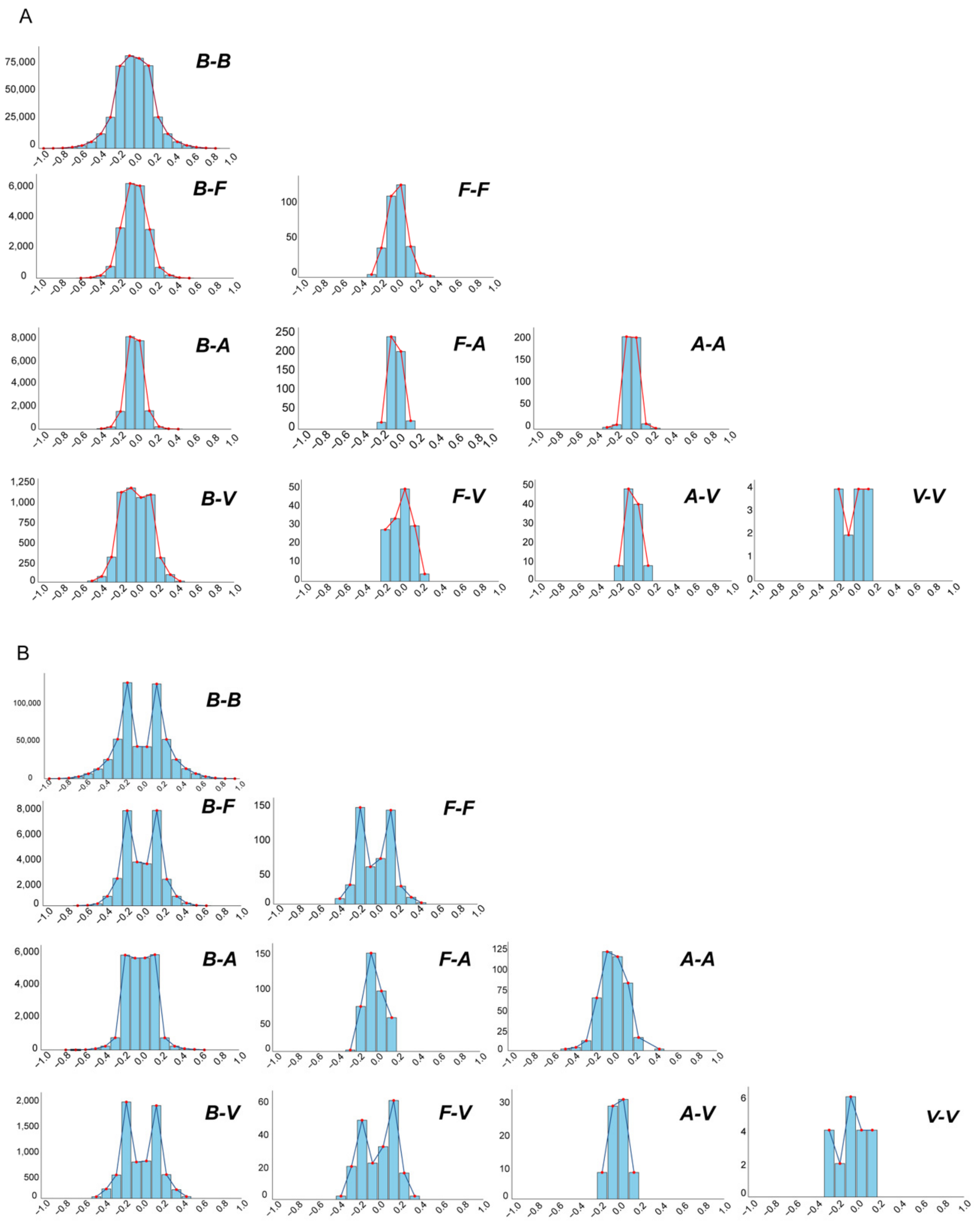

Supplementary Fig. S4. Distribution of SparCC correlation coefficients for different multi-kingdom combinations between two subtypes

(A) Distribution of SparCC correlation coefficients for different multi-kingdom combinations in Subtype C1. The correlation coefficients were normalized to a range of [-1, 1] and grouped into intervals of 0.1. The y-axis indicates the frequency of occurrences of a particular multi-kingdom combination within each interval. The red

lines connect the midpoints at the top of each bar to highlight the distribution trend. (B) Distribution of SparCC correlation coefficients for different multi-kingdom combinations in Subtype C2. The correlation coefficients were normalized to a range of [-1, 1] and grouped into intervals of 0.1. The y-axis indicates the frequency of occurrences of a particular multi-kingdom combination within each interval. The red lines connect the midpoints at the top of each bar to highlight the distribution trend.

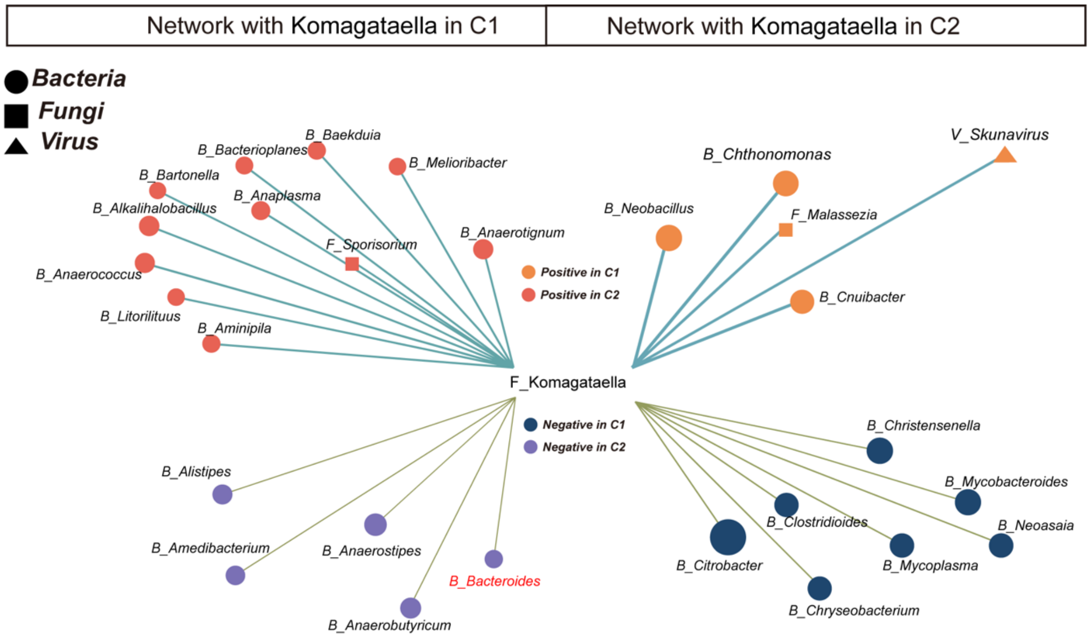

Supplementary Fig. S5. Network with *Komagataella* between two subtypes

The left side of the image shows the correlation of *Komagataella* in subtype C1, while the right side shows the correlation of *Komagataella* in subtype C2.
